# Pre-Hospital Delay and Its Contributing Factors in Patients with ST-Elevation Myocardial Infarction; a Cross sectional Study

**Published:** 2019-05-29

**Authors:** Hamidreza Poorhosseini, Mohammad Saadat, Mojtaba Salarifar, Seyedeh Hamideh Mortazavi, Babak Geraiely

**Affiliations:** 1 Interventional Cardiology Department, Tehran Heart Center, Tehran University of Medical Sciences, Tehran, Iran.; 2 Cardiology Department, Tehran Heart Center, Tehran University of Medical Sciences, Tehran, Iran.

**Keywords:** ST-elevation myocardial infarction, myocardial infarction, STEMI, time-to-treatment Cite

## Abstract

**Introduction:**

The outcome of ST-elevation myocardial infarction (STEMI) is significantly influenced by the total tissue ischemic time. In spite of efforts for reducing the in-hospital delay by full-time provision of primary percutaneous coronary intervention (P-PCI) in the 24/7 program, pre-hospital delay still persists. As a first report in Iran, we aimed to assess the duration of pre-hospital delay and its contributing factors in STEMI patients in the P-PCI era.

**Methods:**

The present cross-sectional study evaluated 2103 STEMI patients who underwent primary PCI from 2016 to 2018. Demographic, personal and socioeconomic factors, index event characteristics, past medical history, pain onset and door times of patients were recorded and independent factors of pre-hospital delay were calculated.

**Results:**

Median (IQR) of pain to door (P2D) time was 279 (120-630) minutes. In multivariate analysis, female gender [Beta=0.064 (95%CI: 0.003-0.125); p=0.038], being uneducated [Beta=0.213 (95%CI: 0.115-0.311); p<0.001], the onset of chest pain between 00:00 to 6:00 [Beta=0.130 (95%CI: 0.058-0.202); p<0.001] or 7:00 to 12:00 [Beta=0.119 (95%CI: 0.049-0.190); p=0.001], self-transportation [Beta=0.098 (95%CI: 0.015-0.181); p=0.020] or referral from another hospital [Beta=0.253 (95%CI: 0.117-0.389); p<0.001], atypical chest pain [Beta=0.170 (95%CI: 0.048-0.293); p=0.006], history of hypertension [Beta=0.052 (95%CI: 0.002-0.102); p=0.041], and opium abuse [Beta=0.076 (95%CI: 0.007-0.146); p=0.031] were associated with a significantly higher log(P2D), while history of CABG was associated with shorter P2D.

**Conclusion:**

Our study showed that P2D is still very high in Iran and revealed the high-risk groups associated with longer P2D. Effective actions should be implemented to increase the public awareness about the symptoms of STEMI, and the importance of immediate appropriate help-seeking.

## Introduction:

Ischemic heart disease is still the most common cause of death worldwide (1-3). Several studies have shown that the morbidity and mortality of patients with ST-elevation myocardial infarction (STEMI) is significantly influenced by the total tissue ischemic time, which consists of pre-hospital and/or in-hospital delays (4-7). High expenditure strategies like primary percutaneous coronary intervention (P-PCI) for STEMI and early invasive strategy for Non-STEMI are developed to reduce the in-hospital component of ischemic time; while a huge amount of golden time is lost in the pre-hospital phase. Efforts have been made in different countries to reduce the total ischemic time.

While in-hospital delay has been reduced in many countries, even developing ones (8, 9), only developed countries have been able to reduce the pre-hospital delay by focusing on total ischemic time through increasing the general population’s awareness via public educational programs in social media (10, 11).

Due to the implementation of full-time (24/7) provision of P-PCI services in our country by the ministry of health and medical education, the in-hospital delay has been reduced in recent years (12). However, as long as the pre-hospital delay remains too long, the benefits of 24/7 P-PCI will be limited. 

There is no large-scale study evaluating the accurate duration of pre-hospital delay in STEMI patients in our country. Given this lack of information, we aimed to assess the duration of prehospital delay and it’s contributing factors in STEMI patients undergoing P-PCI.

## Methods:

***Study design and setting***

The present cross-sectional study, we enrolled 2407 consecutive STEMI patients who underwent P-PCI between January 2016 and December 2018, in a tertiary cardiac center (Tehran Heart center) (13), Tehran, Iran. The hospital’s local review board and Ethics Committee approved of the study protocol (Ethics number: IR.TUMS.MEDICINE.REC.1397.954).

***Participants***

The study population consisted of all STEMI patients who were referred to the mentioned Hospital during the study period and underwent P-PCI. Patients were excluded if the STEMI had occurred in the hospital (n=23). In addition, patients with missed data on pain or door times were excluded from the analysis (n=281). Finally, 2103 STEMI patients were included.

***Data Gathering***

Data on the patients’ demographic information, personal and socioeconomic factors, marital status, educational level, ethnicity, place of longest stay, insurance type, physical activity level, mode of transfer to hospital, pain characteristics, pain onset time, door times, cardiovascular risk factors, and patients’ past medical history, as well as the infarct related artery were extracted from ischemic heart disease, angiography and angioplasty registries of the hospital, which have been described in detail before (14). 

Physical activity level was defined as high in professional athletes, intermediate in those who do usual daily activities and low in patients with the least or lack of physical activity. 

***Statistical analysis***

Continuous variables were presented as mean ± standard deviations (SDs) if they assumed normal distributions and as medians (25^th^ – 75^th^ interquartile ranges: IQR) if they failed to assume normal distributions. Discrete variables were presented as numbers (percentages). P2D was compared between groups using Mann–Whitney test and Kruskal–Wallis test as appropriate. The predictors exhibiting a borderline statistical relationship with pain to door time in the univariate analysis (P ≤ 0.15) were taken for a multivariate logistic regression analysis to investigate their independence. Backward elimination regression analysis was used to remove insignificant variables and log(P2D) was considered as a dependent variable. A P ≤ 0.05 was considered statistically significant. All the statistical analyses were conducted using IBM SPSS Statistics for Windows, version 24.0 (IBM Corp, Armonk, NY, USA).

## Results:

***Baseline characteristics of participants***

2407 consecutive patients were studied out of which 23 cases were excluded due to occurrence of STEMI within the hospital and 281 were excluded because of missed data on pain or door times. Finally, 2103 STEMI patients with the mean age of 59.4911.79 years were enrolled for analysis (76.4% male). Table 1 and 2 summarize the baseline characteristics of studied patients. 94.3% of the patients were married, 79.0% had a diploma or university level education, 77.1% were of Fars ethnicity. Self-transport was the most common form of transfer (86%). Table 3 shows the Index Event’s characteristics. Median (IQR) P2D time of cases was 279 (120-630) minutes.

***Contributing factors of P2D delay (univariate analysis)***

The results of univariate analysis are presented in table 1-3. Based on these analyses female gender was associated with longer median of P2D time (p<0.001) and higher educational level was associated with shorter P2D time (p<0.001). Age had a significant relationship (r=0.036, p=0.095) with log(P2D), while the association was insignificant for BMI (r=0.004, p=0.865).

The P2D time was significantly shorter in those who were transferred to the hospital by EMS (p<0.001). Despite the presence of some meaningful patterns, statistical significance was not observed regarding marital status (p=0.137) and physical activity status (p=0.507).

Description of symptoms as atypical or typical chest pain (p=0.005) and also epigastric pain (p=0.007) was significantly associated with longer P2D. In addition, the history of diabetes (p=0.029) and hypertension (p=0.004) were associated with longer P2D. Although P2D was not different among those with and without the history of coronary stenting (p=0.924), the history of coronary artery bypass graft (CABG) was associated with shorter P2D with borderline significance [191.0 (97.50-425.50) vs. 280.0 (120.0-630.0), p=0.085]. 

***Contributing factors of P2D delay (multivariate analysis)***

After nine steps of the backward elimination method, eight variables remained in the final model (table 4). Female gender (Beta-Coefficient: 0.064, 95%CI: 0.003 - 0.125, p= 0.038), being uneducated (Beta: 0.213, 95%CI: 0.115 - 0.311, p<0.001), the onset of chest pain in 00:00 to 6:00 (Beta: 0.130, 95%CI: 0.058 - 0.202, p<0.001) or 7:00 to 12:00 (Beta: 0.119, 95%CI: 0.049 - 0.190, p=0.001), self-transportation (Beta: 0.098, 95%CI: 0.015 - 0.181, p=0.020) or referral from another hospital (Beta: 0.253, 95%CI: 0.117 - 0.389, p<0.001), description of symptoms as atypical chest pain (Beta: 0.170, 95%CI: 0.048 - 0.293, p=0.006), history of hypertension (Beta: 0.052, 95%CI: 0.002 - 0.102, p=0.041), and opium abuse (Beta: 0.076, 95%CI: 0.007 - 0.146, p=0.031) were associated with longer P2D and the history of CABG (Beta: -0.124, 95%CI: -0.252 - -0.004, p=0.048) was associated with shorter P2D time.

## Discussion:

Based on the findings of the present study, female gender, being uneducated, the onset of chest pain in 00:00 to 6:00 or 7:00 to 12:00, self-transportation or referral from another hospital, description of symptoms as atypical chest pain, history of hypertension and opium abuse were associated with longer P2D while history of CABG was associated with shorter P2D time.

Several studies have been performed in different countries to estimate the interval between pain onset and hospital arrival time. Table 5 demonstrates the median of prehospital delay in STEMI patients in various countries. As is evident grossly, developed countries have succeeded in reducing P2D to around 2 hours, while India as a developing country hasn't shown any obvious progress during these years.

Limited studies with small sample sizes have been conducted regarding prehospital delay in Iran (Table 6). As is evident, all of them were performed before implementation of 24/7 program. Except for one study, all of them have small sample sizes and their results are greatly discordant. To the best of our knowledge, this is the first study to evaluate predictors of prehospital delay in Iran in a large population of STEMI patients undergoing P-PCI.

In the current study, using multivariate analysis, pain to door time was found to be significantly higher in female gender, uneducated patients, those with onset of chest pain between 00:00 to 6:00 or 7:00 to 12:00, self-transported patients or individuals who were referred from other hospitals, patients with atypical chest pain and history of hypertension and opium abuse; while history of CABG was associated with shorter pain to door time.

In a study by Noorani et al. (15), prehospital delay has been shown to be associated with long distance from hospital, lower socioeconomic status and using ambulance.

In a study by Moser el al. (11) several factors have been mentioned to be associated with prehospital delay including female gender, older age, worse socioeconomic status, history of angina, having cardiovascular risk factors and poor knowledge of the individual.

In the current study we found that patients with chest pain between 00:00 to 6:00 or 7:00 to 12:00 had higher prehospital delays. On the contrary, patients transferred by EMS and educated individuals had lower pain to door time. Infarct related artery had no significant effect in pain to door time in our study population. Our findings are in line with those of Peng et al. (16) who assessed 1088 STEMI patients. They demonstrated that prehospital delay was negatively correlated with high educational level, previous history of MI, transportation by ambulance, onset of pain during the daytime (6:00-18:00) and anterior and posterior MI.

In our study, the level of education was negatively correlated with pain to door time. Similar to our work, the study of Heo et al. (17), reported a pain to door time of 144, 76 and 68 minutes in STEMI patients with low, moderate and high educational levels, respectively. In MEDEA Study (18) on 486 acute MI patients, prehospital delay was higher in patients with low MI-knowledge. They also found that patients with atypical symptoms had higher prehospital delays, which corresponds to our findings. 

**Table 1:** Demographic and socioeconomic characteristics

**Variables**	**Number (%)**	**Prehospital Delay (minute)**		**p**
		**Median**	**IQR 25% - 75%**	
**Gender**				
Male	1607(76.4)	255.00	110.00 - 595.00	<0.001
Female	496 (23.6)	337.00	160.00 - 790.00	
**Marital status**				
Married	1983 (94.3)	270.50	117.00 - 619.75	0.137
Single	17 (0.8)	314.00	122.50 - 625.50	
Divorced	18 (0.9)	227.50	83.00 - 1020.25	
Widowed	85 (4.0)	349.00	172.50 - 858.00	
**Education**				
University	279 (13.4)	206.00	90.00 - 465.00	<0.001
High school diploma	1362 (65.6)	265.00	115.00 - 606.75	
Elementary education	175 (8.4)	310.00	136.00 - 662.00	
Uneducated	260 (12.5)	400.50	184.25 - 1014.00	
**Ethnicity**				
Fars	1622 (77.1)	270.00	117.00 - 603.75	0.267
Turk	299 (14.2)	303.00	130.00 - 730.00	
Other	182 (8.7)	318.00	120.00 - 806.00	
**Longest linger**				
Tehran	1507 (71.7)	260.00	110.00 - 603.25	0.011
Main Cities	162 (7.7)	355.00	141.00 - 732.50	
Small Cities	161 (7.7)	312.00	140.25 - 689.75	
Village	273 (13.0)	300.00	138.00 - 659.50	
**Insurance**				
Social security	843 (40.1)	273.00	120.00 - 648.50	0.609
Health facilities	704 (33.5)	285.00	120.25 - 625.75	
Other companies	390 (18.5)	289.00	120.00 - 600.00	
No insurance	166 (7.9)	239.00	90.75 - 601.50	
**Physical activity level**				
High	49 (2.3)	206.00	128.75 - 574.00	0.507
Intermediate	1986 (94.4)	279.00	118.50 - 624.50	
Low	68 (3.2)	310.00	129.00 - 790.00	

**Table 2:** Past medical history of the patients

**Variables**	**Number (%)**	**Prehospital Delay (minute)**		**p**
		**Median**	**IQR 25% - 75%**	
**Diabetes mellitus**				
Yes	657 (31.2)	309.50	123.25 - 629.25	0.029
No	1446 (68.8)	264.00	115.25 - 628.00	
**Hypertension**				
Yes	852 (40.5)	315.50	130.75 - 670.00	0.004
No	1251 (59.5)	250.00	110.00 - 596.25	
**Hyperlipidemia**				
Yes	880 (41.8)	266.50	116.25 - 612.00	0.383
No	1223 (58.2)	285.50	120.00 - 640.25	
**Smoking**				
Yes	739 (35.1)	252.00	111.50 - 612.00	0.235
No	1364 (64.9)	290.00	120.00 - 649.00	
**Opium abuse**				
Yes	303 (14.4)	340.00	133.00 - 663.00	0.081
No	1800 (85.6)	270.00	117.00 - 618.00	
**Family history of cad**				
Yes	354 (16.8)	253.00	102.00 - 607.50	0.066
No	1749 (83.2)	285.00	120.00 - 642.00	
**Cerebrovascular event**				
Yes	78 (3.7)	266.00	111.00 - 547.50	0.852
No	2025 (96.3)	279.00	120.00 - 630.00	
**Chronic kidney disease**				
Yes	46 (2.2)	350.50	157.00 - 679.50	0.324
No	2057 (97.8)	274.00	119.75 - 627.75	
**History of cabg**				
Yes	77 (3.7)	191.00	97.50 - 425.50	0.085
No	2026 (96.3)	280.00	120.00 - 630.00	
**History of myocardial infarction**				
Yes	153 (7.3)	330.00	125.00 - 890.00	0.053
No	1950 (92.7)	270.00	119.00 - 610.00	
**History of coronary stenting**				
Yes	122 (5.8)	266.00	110.00 - 733.75	0.924
No	1981 (94.2)	279.50	120.00 - 621.25	
**Infarct related artery**				
LAD	1144 (54.4)	270.00	120.00 - 642.50	0.621
LCX	297 (14.1)	310.00	119.25 - 583.25	
RCA	612 (29.1)	287.00	120.00 - 645.75	
SVG	50 (2.4)	162.00	98.75 - 898.00	

Hx, history; CAD, coronary artery disease; CABG, coronary artery bypass graft; LAD, left anterior descending; LCX, left circumflex; RCA, right coronary artery; SVG, saphenous vein graft.

**Table 3:** Index event’s characteristics

** Index**	**Number**	**Prehospital Delay (minute)**		**p**
		**Median**	**IQR 25% - 75%**	
**Mode of transfer**				
Ambulance	196 (9.3)	209.00	91.25 - 458.00	<0.001
Self-Transport	1808 (86.8)	280.00	120.00 - 650.00	
Referral	99 (4.7)	364.00	208.50 - 684.00	
**Pain onset time**				
0 to 6	554 (26.4)	345.00	112.00 - 872.00	<0.001
7 to 12	617 (29.4)	324.00	135.00 - 677.50	
13 to 18	519 (24.7)	264.00	120.00 - 462.00	
19 to 24	412 (19.6)	205.00	106.25 - 549.50	
**Pain description**				
Typical Chest Pain	1984 (94.3)	270.00	117.00 - 613.00	0.005
Atypical Chest Pain	84 (4.0)	488.00	176.00 - 895.75	
No Chest Pain	35 (1.7)	307.00	99.00 - 705.00	
**Pain duration**				
>30 min	1183 (56.3)	300.00	120.00 - 663.00	0.022
11-30 min	801 (38.1)	248.00	112.50 - 581.50	
1-10 min	84 (4.0)	324.00	142.00 - 847.00	
No Chest Pain	35 (1.7)	307.00	99.00 - 705.00	
**Back pain**				
Yes	26 (1.2)	377.50	149.25 - 904.50	0.146
No	2077 (98.8)	275.50	119.00 - 620.25	
**Epigastric pain**				
Yes	277 (13.2)	335.50	144.25 - 701.75	0.007
No	1826 (86.8)	270.00	115.00 - 615.00	
**Jaw pain**				
Yes	14 (0.7)	221.50	131.25 - 553.75	0.640
No	2089 (99.3)	278.50	120.00- 630.00	
**Left precordial pain**				
Yes	1022 (48.6)	265.00	113.50 - 612.00	0.162
No	1081 (51.4)	289.00	120.00 - 645.00	
**Retro-sternal pain**				
Yes	1400 (66.6)	270.00	115.00 - 630.00	0.223
No	703 (33.4)	285.00	127.50 - 616.00	
**Right precordial pain**				
Yes	11 (0.5)	345.00	205.00 - 610.00	0.495
No	2092 (99.5)	276.00	119.50 - 630.00	
**Arm & shoulder pain**				
Yes	71 (3.4)	227.50	140.25 - 574.00	0.815
No	2032 (96.6)	279.50	117.25 - 630.75	

**Table 4:** Multivariate analysis for prediction of log(p2d)

**Variable**	**Beta Coefficient**	**95% Confidence Interval**		**p**
		**Lower**	**Upper**	
**Gender**				
Male	-	-	-	-
Female	0.064	0.003	0.125	0.038
**Education**				
University	-	-	-	-
High school diploma	0.070	-0.002	0.143	0.058
Elementary education	0.082	-0.025	0.189	0.135
Uneducated	0.213	0.115	0.311	<0.001
**Pain Onset Time**				
19 to 24	-	-	-	-
13 to 18	0.043	-0.030	0.116	0.243
7 to 12	0.119	0.049	0.190	0.001
0 to 6	0.130	0.058	0.202	<0.001
**Mode of Transfer**				
Ambulance	-	-	-	-
Self-Transfer	0.098	0.015	0.181	0.020
Referral	0.253	0.117	0.389	<0.001
**Pain Description**				
Typical Chest Pain	-	-	-	-
Atypical Chest Pain	0.170	0.048	0.293	0.006
No Chest Pain	0.066	-0.121	0.253	0.491
**Hypertension**				
No	-	-	-	-
Yes	0.052	0.002	0.102	0.041
**Opium**				
No	-	-	-	-
Yes	0.076	0.007	0.146	0.031
**CABG**				
No	-	-	-	-
Yes	-0.124	-0.252	-0.004	0.048

CABG: coronary artery bypass graft.

**Table 5:** Median of prehospital delay in ST-elevation myocardial infarction patients in various countries according to published reports

**Country**	**Prehospital delay (minute)**	**Year**
United Stated (10, 19)	290	1990
	84 in males 121 in females	2002
	59 in males 81 in females	2006
Denmark (20)	125	1998
Australia and New Zealand (21)	145	2008
South Korea (22)	130	2012
India (23, 24)	310 290	2003 2016

**Table 6:** Iranian studies on prehospital delay

**Study**	**Year**	**City**	**Number** *	** ACS forms**	**P2D**
Momeni (25)	2011	Rasht	162	STEMI	120
Khosravi (26)	2011	Isfahan	103	STEMI	255
Farshidi (27)	2012	Hormozgan	227	STEMI & NSTEMI	N/A
IPACE2 (28)	2012	Tehran, Mashhad, Isfahan, Shiraz, Tabriz	1997	UA & NSTEMI & STEMI	265
Taghadosi (29)	2013	Kashan	117	STEMI & NSTEMI	129

* : number of patients, P2D: pain to door time (minutes), ACS: acute coronary syndrome.

Our study showed that P2D is still very long in Iran and revealed the high-risk groups associated with longer P2D. We assume that effective actions should be implemented to increase the general population’s knowledge about the presentations of acute MI in order to decrease the time to seek treatment.

***Limitations and Strengths***

Being single-centered and retrospective design of the current work can be considered as our study limitations. We had missing values in pain or door times in 281 patients and thus we excluded them from the final analysis. We had no information regarding the patients’ place of living and could not retrieve the data on their distance from the hospital. Meanwhile, the present study is the largest study that has been done to evaluate P2D in Iranian patients and the first study that has published after starting the 24/7 program. Unlike IPACE2 study, only STEMI patients, for whom P2D is applicable and can be defined, were included in our study.

## Conclusion:

In the present study female gender, transfer via vehicles other than ambulance, atypical chest pain, low level of education, late night and morning onset of pain, history of hypertension and opium abuse were associated with higher prehospital delay while history of CABG was associated with shortened P2D. 
